# Comparison of artificial neural network and logistic regression models for prediction of mortality in head trauma based on initial clinical data

**DOI:** 10.1186/1472-6947-5-3

**Published:** 2005-02-15

**Authors:** Behzad Eftekhar, Kazem Mohammad, Hassan Eftekhar Ardebili, Mohammad Ghodsi, Ebrahim Ketabchi

**Affiliations:** 1 Department of Neurosurgery, Sina Hospital, Tehran University, Tehran, Iran; 2 Department of Biostatistics and Epidemiology, Faculty of Public Health, Tehran University, Tehran, Iran; 3 Department of Public Health, Faculty of Public Health, Tehran University, Tehran, Iran

## Abstract

**Background:**

In recent years, outcome prediction models using artificial neural network and multivariable logistic regression analysis have been developed in many areas of health care research. Both these methods have advantages and disadvantages. In this study we have compared the performance of artificial neural network and multivariable logistic regression models, in prediction of outcomes in head trauma and studied the reproducibility of the findings.

**Methods:**

1000 Logistic regression and ANN models based on initial clinical data related to the GCS, tracheal intubation status, age, systolic blood pressure, respiratory rate, pulse rate, injury severity score and the outcome of 1271 mainly head injured patients were compared in this study. For each of one thousand pairs of ANN and logistic models, the area under the receiver operating characteristic (ROC) curves, Hosmer-Lemeshow (HL) statistics and accuracy rate were calculated and compared using paired T-tests.

**Results:**

ANN significantly outperformed logistic models in both fields of discrimination and calibration but under performed in accuracy. In 77.8% of cases the area under the ROC curves and in 56.4% of cases the HL statistics for the neural network model were superior to that for the logistic model. In 68% of cases the accuracy of the logistic model was superior to the neural network model.

**Conclusions:**

ANN significantly outperformed the logistic models in both fields of discrimination and calibration but lagged behind in accuracy. This study clearly showed that any single comparison between these two models might not reliably represent the true end results. External validation of the designed models, using larger databases with different rates of outcomes is necessary to get an accurate measure of performance outside the development population.

## Background

In recent years, outcome prediction studies have become the avante garde in many areas of health care research, especially in critical care and trauma. However acceptable models for outcome prediction have been difficult to develop [1]. According to Wyatt and Altman, to be useful, a predictive model must be simple to calculate, have an apparent structure and be tested in independent data sets with evidence of generality [2]. While this is a high standard, availability and popularity of portable computers, deprioritize the need for simplicity of the model and having an apparent structure.

Artificial neural networks (ANNs) are mathematical constructs modeled on interconnection of nodes (neurons) giving a loose association with the animal nervous system. [3]

ANNs employ nonlinear mathematical models to mimic the human brain's own problem-solving process. Just as humans apply knowledge gained from past experience to new problems or situations, a neural network takes previously solved examples to build a system of "neurons" that makes new decisions, classifications, and forecasts. [4]

ANNs are complex and flexible nonlinear systems with properties not found in other modeling systems. These properties include robust performance in dealing with noisy or incomplete input patterns, high fault tolerance, and the ability to generalize from the input data [5]. Neural networks excel at applications where pattern recognition is important, and precise computational answers are not required, such as forecasting weather, stock predicting, or speech recognition [6]. Reports in medical literature suggest that ANN models are applicable in diagnosing diseases such as myocardial infarction [7,8] pulmonary emboli detection [9], gastrointestinal hemorrhage [10], waveform analysis of EKGs [11], EEGs [12,13], and radiographic images [14]. ANNs have also been successfully applied in clinical outcome prediction of trauma mortality [1,15], surgical decision making on traumatic brain injury patients [16], recovery from surgery [17,18], outcome in pediatric meningococcal disease [19] and transplantation outcome [20].

Lang EW et al have compared ANN with Logistic Regression in prediction of outcome after severe head injury and concluded that the differences in the results obtained with the two models were negligible [21].

Almost all of the published articles indicate that the performance of ANN models and logistic regression models have been compared only once in a dataset and the essential issue of internal validity (reproducibility) of the models has not been addressed.

The objective of this study was to compare the performance of ANN and multivariate logistic regression models for prediction of mortality in head trauma based on initial clinical data and whether these models are reproducible. We used different variables even if they were interdependent.

## Methods

### Study population

Among 8452 trauma patients' records admitted to the emergency departments of six major university hospitals in Tehran from 23 August 1999 to 22 September 2000, the records of 1271 patients whose main trauma was head injury, were selected for this study. The selection of head trauma as the main trauma was based on the definition of principal diagnosis in the Uniform Hospital Discharge Data Set (UHDDS). It defines the principal diagnosis as "that condition established after study to be chiefly responsible for occasioning the admission of the patient to the hospital for care". For making determination of the main trauma more practical in the case of ambiguity, hospitalization in the neurosurgical ward was used as an additional guideline. The database was based upon the trauma data registry program began in 1996 in Trauma Research Center, Sina Hospital, a hospital affiliated with the Tehran University of Medical Sciences [22,23]. The study population for this study was comprised of all trauma victims who had been admitted in one of the hospitals for more than 24 hours during the data-gathering period. For dead patients, this time limitation was disregarded, that is, records of all dead patients who were referred to these hospitals were included in the study. We have excluded those transferred to other hospitals or with related missing values. Structured, closed-question data checklists were used for the data gathering process. Three major categories of injury-related information were collected, that is, demographic data, pre-hospital data (if they were available) and in-hospital data. Hospital related data included: vital signs, Glasgow Coma Scale (GCS), Abbreviated Injury Scale (AIS-90 [24]), clinical findings in accordance with the International Classification of Diseases 10^th ^revision (ICD-10) as well as the outcome of the patients. Data collection was conducted by a group of trained physicians who had completed special training courses to become familiar with the process of extracting Abbreviated Injury Score (AIS-90) codes and filling out the relevant questionnaires. For quality control (QC) purposes each hospital had a physician, who was responsible for overseeing the data gathering process. In the intubated patients GCS were calculated according to the other portions of the scale by this physician. Finally, a medical practitioner examined all the checklists in order to evaluate and amend them if deemed necessary based on pre-arranged and fixed protocols.

Since we were trying to build and compare models for prediction of outcome mainly based on the initial clinical data, only data related to the GCS, tracheal intubation status, age, systolic blood pressure (SBP), respiratory rate(RR), pulse rate(PR), injury severity score (ISS)(upon admission) and outcome were used in our study. In order to prepare the data for the Neural Network software and to enhance the reliability of the data, three variables of systolic blood pressure, respiratory rate and pulse rate were transformed to dichotomous(1,0) variables. Low systolic blood pressure was defined according to the following cutoff points: up to 5 years of age, less than 80 mmHg; and 5 years of age or older, less than 90 mmHg. Respiratory rate of 35 per min and pulse rate of 90 per min were selected as limits for definition of tachypnea and tachycardia. Other variables including GCS, age, systolic blood pressure (SBP) and injury severity score (ISS) variables were also converted from decimal (Base 10) to binary (Base 2). This conversion was carried out in order to render the input data suitable for processing by our ANN software with its default settings. The data and the data format were similar for both ANN and logistic regression models.

### Development of logistic regression models

The dataset was divided randomly into two sets, one set of 839 cases (66% of the whole dataset) for training and 432 cases for testing the model. A model was built using a training set with logistic regression. GCS, tracheal intubation status(dichotomous), age, SBP(dichotomous), RR(dichotomous), PR(dichotomous) and ISS were the independent variables and the outcome (death/survival) was the dependent variable. The logistic regression analyses were performed using Intercooled STATA for windows, Version 6 (STATA Corp., College Station, TX) "logistic" default options.

The built logistic model was tested using the testing dataset (432 cases). These steps (randomized division of dataset and regression analysis considering the same variables) were repeated 1000 times. This resulted in 1000 pairs of training and testing datasets (2/3 and 1/3 of the original dataset, respectively) which were saved for further processing by the neural networking.

### Development of ANN models

**Figure 1:**
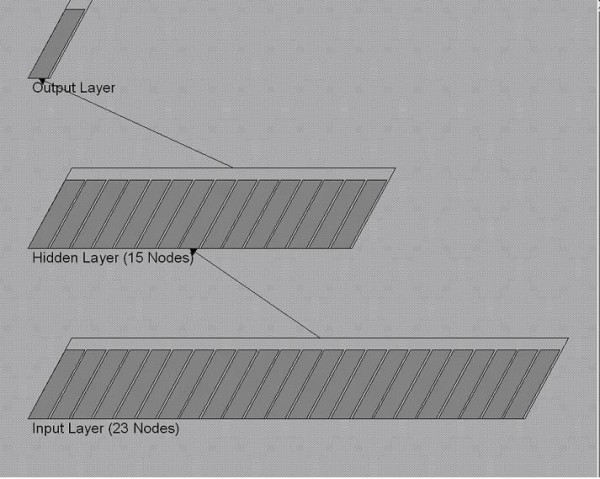
Diagrammatic representation of Artifical Neural Network ANN) structure with 23 input nodes in the input layer, 15 nodes in the hidden layer and one node in the output layer.

The ANN used in this study was a standard feed-forward, back-propagation neural network with three layers: an input layer, a hidden layer and an output layer. The input layer consisted of 23 input neurons, the hidden layer consisted of fifteen hidden neurons, and the output layer consisted of one output neuron (Fig. 1). The learning rate and momentum for network training were set respectively to 0.25 and 0.9 and the models were run until a minimum average squared error < 0.063 was obtained. The number of the network layers, hidden neurons and the stopping criteria were determined through trial-and-errors process because no commonly accepted theory exists for predetermining the optimal number of neurons in the hidden layer [25].

The training and testing datasets were the same as those were used with regression models, thus there was an ANN and a logistic model for each training and testing dataset.

PDP++ version 3.1 was used for the artificial neural network analysis as it has a powerful built-in scripting language and is freely available. This software can be downloaded from .

### Comparison of model performance

Discrimination and calibration (goodness of fit) were both measured. Discrimination refers to the ability of a model to distinguish those who die from those who survive. A perfectly discriminating model would assign a higher probability of death to all cases that died than to any case that survived. The discriminatory power of the models was analyzed using the area under the receiver operating characteristic curves (ROC-A(z)). ROC curves were constructed by plotting true positives (patients who died and whom the model predicted as dying [i.e. sensitivity]) versus the false positive fraction (fraction of the patients who lived and were incorrectly classified as dying [i.e. (1 – specificity)]).

A ROC-A(z) value of one corresponds to a test that perfectly separates two populations, whereas a ROC-A(z) value of 0.5 corresponds to a perfectly useless test that performs no better than chance.

The relative calibration of the models, that is, how accurately the models predicted over the entire range of severity, was compared using Hosmer-Lemeshow (HL) statistics. The HL statistics is a single summary measure of the calibration and is based on comparing the observed and estimated mortality for patients grouped by estimated mortality. The resulting statistic follows a chi-squared distribution, with degrees of freedom equal to two less than the number of groups (10 in this study). The smaller the HL statistics, the better the fit, with a perfectly calibrated model having a value of zero.

A probability cut point of 0.5 was used to classify observations as events or nonevents. The overall accuracy ([true positive+true negative]/total) of the final model was determined by comparing the predicted values with the actual events.

For each of one thousand pairs of ANN and logistic models (trained and tested on the same datasets), these indices (area under the ROC curves, HL statistics and accuracy rate) were calculated and compared using paired T-tests (P < 0.05).

All the statistical analyses were performed using Intercooled STATA for windows, version 6 STATA Corp., College Station, TX) and its downloadable add-on ado files.

Some of the scripts used both for the STATA and PDP++ and the designed neural networks can be downloaded from the site of the Tehran University of Medical Sciences . Further details are available from the corresponding author.

## Results

Table 1 shows clinical characteristics of the dataset. The mean age of the study population was (28.5 ± 19) years. 76% of our patients were male and the overall mortality rate was 7.5%. 7.5% of the patients had GCS < 8.

**Table 1:** General Characteristics of the Dataset

GCS	13.5 ± 3
Sex	76% Male
ISS	9.5 ± 14
SBP	117 ± 23
RR	19 ± 7
PR	86 ± 17
Intubated	2%
Age	28.5 ± 19
Mortality	7.5%

GCS = Glasgow Coma Scale; ISS = Injury Severity Score; SBP = Systolic Blood Pressure; RR = Respiratory Rate; PR = Pulse Rate

As is seen in Table 2 ANN significantly outperformed logistic models in both senses of discrimination and calibration, although from the standpoint of accuracy (cutoff point 0.5), logistic models were superior to ANN models. In 77.8% of cases the area under the ROC curve and in 56.4% of cases the HL statistics for the neural network model were superior to that for the logistic model. In 68% of cases the accuracy of the logistic model was superior to the neural network model.

**Table 2:** Results of Comparing 1000 pairs of Artificial Neural Network (ANN) and Logistic Regression (LR) models

	LR(95% Confidence Interval)	ANN(95% Confidence Interval)	P <
Area under ROC curve	.9538 (.9527 – .9549)	.9646 (.9627 – .9665)	0.0000
H-L Statistics	53.13 (46.04 – 60.23)	41.51 (32.92 – 50.11)	0.015
Accuracy Rate	96.37 (96.32 – 96.42)	95.09 (94.93 – 95.24)	0.0000

The confidence intervals of the all the measures show higher variation in the ANN models results.

## Discussion

Prognostic evaluation of the patients without CT scan findings may have limited applicability considering the availability of CT scans in the majority of the trauma centers. Nevertheless, the idea of prognostic models based on initial clinical data at admission is still worth trying and seems to be of practical value in some situations. Although omission of CT data weakens our armamentarium significantly, patients without paraclinical and imaging data make the real trauma scenes at which medical staff should have an evaluation. Actually as neurosurgeons we have initial clinical judgments based on our growing experiences and in many situations they prove to be true. We can expect computers do similar things and help us. In fact, vague situations like what we see in primary evaluation of head trauma patients are where ANN may prove to be superior to traditional linear modeling. This is one of the reasons that although many paraclinical and imaging factors are known to be of significant predictive value in the outcome of the head trauma patients, this study only used clinical measures which are simply available to a physician in the emergency department.

### Study limitations

Calculating ISS and AIS are not simple tasks and needs training. This can reduce the practicability of the results. The pupillary size and reactivity are one of the clinical signs with prognostic value that were not considered in the study due to defect of the main database in this regard.

As is seen the mortality rate and the percentage of patients with GCS < 8 are the same (7.5%). This is a coincidence, but emphasizes the need for further studies in the populations with different rates of outcome.

The mean GCS was 13.5 ± 3. The standard deviation is over the top score of GCS (15) and means that the GCSs were skewed towards higher levels of consciousness.

Not considering the exact time interval between head trauma and admission which is simply available for the medical staff is one of the weak points of this study.

The lower intubation rate in this population study related to the distribution of the GCS may show the lower quality of the pre-hospital care prevailing in the hospitals selected for this study. This may render the reproduction of the results using the same network (Downloadable from ) somewhat difficult in other situations where pre-hospital care services are more advanced.

Using ISS as an independent variable underlines the role of general trauma in the models used for this study. This was unavoidable, because the original dataset had been a subset of general trauma patients.

### Comparison of two models

Currently, the logistic regression and the artificial neural networks are the most widely used models in biomedicine, as measured by the number of publications indexed in Pubmed as attested by 45646 cases for the logistic regression and 8015 for the neural network.

Logistic regression is a commonly accepted statistical tool, which can generate excellent models. Its popularity may be attributed to the interpretability of model parameters and ease of use, although it has limitations. For example, logistic regression models use linear combinations of variables and, therefore, are not adept at modeling grossly nonlinear complex interactions as has been demonstrated in biologic and complex epidemiologic systems.

Not withstanding its limitations, neural networks are appealing for a number of reasons, namely; they seem to "learn" without supervision, they can be created by workers with very little mathematical model building experience, and software for building neural networks is now readily available. Neural networks have perhaps a special appeal to the medical community because of their superficial resemblance to the human brain (a structure with which most physicians are comfortable), and seem to promise "prediction" without the difficulties associated with use of mathematics.

ANNs are rich and flexible nonlinear systems that show robust performance in dealing with noisy or incomplete data and have the ability to generalize from the input data. They may be better suited than other modeling systems to predict outcomes when the relationships between the variables are complex, multidimensional, and nonlinear as found in complex biological systems.

The difficulty in developing models using artificial neural networks is that there are no set methods for constructing the architecture of the network. The most common type of artificial neural networks is the feed-forward back propagation multiperceptron (used in this study).

Another limitation of neural network models is that standardized coefficients and odds ratios corresponding to each variable cannot be easily calculated and presented as they are in regression models. Neural network analysis generates weights, which are difficult to interpret as they are affected by the program used to generate them [26]. This lack of interpretability at the level of individual variables (predictors) is one of the most criticized features in neural network models [27].

Several early applications of neural networks in medicine reported an excellent fit of the ANN model to a given set of data. The impressive results usually were derived from over fitted models, where too many free parameters were allowed. Linear and logistic regression models have less potential for overfitting primarily because the range of functions they can model is limited.

Neural network models require sophisticated software. The complexity and unfamiliarity of ANN has been a major drawback of this technique so far. However, as palmtop computing becomes increasingly powerful and popular, the complexity of ANNs may become less onerous in real-time clinical settings. [4]

Furthermore, there are some theoretical advantages comparing a predictive ANN model over conventional models such as logistic regression.

One such advantage is that ANN model allows the inclusion of a large number of variables [28]. Another advantage of the neural network approach is that there are not many assumptions (such as normality) that need to be verified before the models can be constructed.

Although, one of the strengths of ANNs is their ability to still find patterns despite missing data, in this study a dataset with no related missing values was used.

Recently the task of comparison between these two models has been addressed from different points of view. Considering the publication bias, several published works in the medical literature have demonstrated the success of the ANN approaches. In a review carried out by Sargent on 28 major studies, ANN outperformed regression in 10 cases (36%), was outperformed by regression in 4 cases (14%) and the 2 methods had similar performance in the remaining cases. Sargent concluded that both methods should continue to be used and explored in a complementary manner. [29]

Gaudart et al. using simulated data, have compared the performance of ANN and linear regression models for epidemiological data and concluded that both had comparable performance and robustness and despite the flexibility of connectionist models (like ANN), their predictions were stable. [30]

This study was primarily designed to compare the performance of an ANN and a multivariable logistic regression analysis with the goal of developing a model for predicting the outcome in head injury and for studying their internal validity (reproducibility). Also setting up a standalone practical model for prediction of mortality in the head trauma patients was a secondary goal of this study.

Using freely downloadable software (PDP++) and making the networks and scripts accessible by the researchers can be perceived as an advantage of this study.

This study showed that ANN models significantly outperformed logistic models in both senses of discrimination and calibration, although lagged behind in accuracy. It is pointed out that the calibration values should be treated with some caution in this study, since according to Hosmer and Lemeshow, [31] in describing the statistic, HL statistic should be used where at least one of the predictor variables is continuous.

This study clearly shows that in a single comparison of these models based on the same data there is 22.2% chance of getting discrimination results contrary to our findings in majority of comparisons. This ratio is 43.6% for calibration and 32% for the accuracy results. These figures are practically important and imply that any single comparison between these two models cannot reliably represent their final performance.

Although considerable efforts, through many trial-and-errors, were made to optimize the design of the network, the designed ANN models could, and should, be further improved. In line with any other predictive models, likewise the findings of this study need to be externally validated. The networks are downloadable and the results can be studied in other study populations with divergent data and different survival ratios.

So far, there is no single algorithm that performs better than all other algorithms for any set of given head injury data. To this end, there is room for much more work to be done before a definite conclusion can be reached.

### Potential clinical use

Should the results be reproducible in other populations, using a simple preprogrammed calculator (or other programmable computing devices) and minimal training of the personnel, this model and similar ones may emerge to be of considerable practical value in triage of the patients. At that time a dedicated instead of general purpose ANN software should be designed for this purpose.

The authors concur with the conclusion arrived by Tu [25] that logistic regression remains the clear choice when the primary goal of model development is to examine possible causal relationships among variables. However, it appears that ANNs or some form of hybrid technique incorporating the best features of both logistic regression and neural network models might lead to development of optimum prediction models for head injured patients.

## Conclusions

In conclusion, this study compared models for the prediction of outcome in head injury using trauma data from hospital registries in Tehran, the data was applied to artificial neural network and multivariable logistic regression analysis. The predictive ability of the artificial neural network model was found to be comparable to that of the logistic regression model. Specifically, the ANN models significantly outperformed logistic models in both senses of discrimination and calibration but lagged behind in accuracy. Although the performance of the models were studied when the models were applied to the different samples of the original population study, external validation is necessary to get an accurate measure of performance outside the development population. Studies using larger databases with different rates of outcomes may further clarify the differences between artificial neural network and logistic regression models in head injury outcome prediction and their clinical implications.

## Competing interests

The author(s) declare that they have no competing interests.

## Authors' contributions

BE carried out the data extraction, performed the analysis and drafted the manuscript. KM supervised the analysis and critically reviewed the statistical viewpoints. HEA, MG and EK supervised the study and participated in its coordination. All authors read and approved the final manuscript.

## Pre-publication history

The pre-publication history for this paper can be accessed here:


